# Performance of a hard X-ray split-and-delay optical system with a wavefront division

**DOI:** 10.1107/S1600577517014023

**Published:** 2018-01-01

**Authors:** Takashi Hirano, Taito Osaka, Yuki Morioka, Yasuhisa Sano, Yuichi Inubushi, Tadashi Togashi, Ichiro Inoue, Satoshi Matsuyama, Kensuke Tono, Aymeric Robert, Jerome B. Hastings, Kazuto Yamauchi, Makina Yabashi

**Affiliations:** a Department of Precision Science and Technology, Graduate School of Engineering, Osaka University, 2-1 Yamada-oka, Suita, Osaka 565-0871, Japan; b RIKEN SPring-8 Center, 1-1-1 Kouto, Sayo-cho, Sayo-gun, Hyogo 679-5148, Japan; c Japan Synchrotron Radiation Research Institute (JASRI), 1-1-1 Kouto, Sayo-cho, Sayo-gun, Hyogo 679-5198, Japan; d Linac Coherent Light Source, SLAC National Accelerator Laboratory, 2575 Sand Hill Road, Menlo Park, CA 94025, USA; e SLAC National Accelerator Laboratory, 2575 Sand Hill Road, Menlo Park, CA 94025, USA

**Keywords:** hard X-ray free-electron laser, split-and-delay optics, multi-crystal optics, X-ray photon correlation spectroscopy

## Abstract

The performance of a hard X-ray split-and-delay optical (SDO) system with a wavefront division scheme was investigated at the hard X-ray free-electron laser facility SACLA. For the wavefront division, beam splitters made of edge-polished perfect Si(220) crystals were employed. We characterized the beam properties of the SDO system, and investigated its capabilities for beam manipulation and diagnostics. First, it was confirmed that shot-to-shot non-invasive diagnostics of pulse energies for both branches in the SDO system was feasible. Second, nearly ideal and identical focal profiles for both branches were obtained with a spot size of ∼1.5 µm in full width at half-maximum. Third, a spatial overlap of the two focused beams with a sub-µm accuracy was achieved by fine tuning of the SDO system. Finally, a reliable tunability of the delay time between two pulses was confirmed. The time interval was measured with an X-ray streak camera by changing the path length of the variable-delay branch. Errors from the fitted line were evaluated to be as small as ±0.4 ps over a time range of 60 ps.

## Abstract 2

The performance of a hard X-ray split-and-delay optical system with a wavefront division scheme has been investigated at SACLA.

## 1. Introduction   

The advent of hard X-ray free-electron lasers (XFELs) with ultra-high intensity, excellent transverse coherence and pulse duration of less than 10 fs has offered new opportunities in revealing ultrafast dynamics in matter at angstrom spatial and femtosecond time scales. New insights into ultrafast phenomena have been obtained *via* an optical pump and XFEL probe technique in which the prompt optical laser pulse excites electrons at a valence level and then the delayed XFEL pulse probes the following dynamics, such as chemical reactions (Kim *et al.*, 2015 ▸), atomic and magnetic ordering/dis­ordering (Dean *et al.*, 2016 ▸) and phonons (Clark *et al.*, 2013 ▸; Trigo *et al.*, 2013 ▸), as a function of the delay time between the pump and probe pulses. X-ray-induced damage processes associated with the core-excitation and following multiple ionization have been studied through an XFEL pump and XFEL probe technique (Inoue *et al.*, 2016 ▸) with an accelerator-based dual XFEL pulse generation (DXPG) method (Hara *et al.*, 2013 ▸). In this case, a typical delay time range is from a few femtoseconds to hundreds of femtoseconds. With an extended delay range up to sub-picoseconds or nano­seconds, spontaneous fluctuations in diverse systems at angstrom to nanometer spatial scales will be unveiled through, for example, an X-ray photon correlation spectroscopy (XPCS; Grübel *et al.*, 2007 ▸; Gutt *et al.*, 2009 ▸) method using XFEL pulse pairs, although these ranges are inaccessible with the accelerator-based DXPG methods (Hara *et al.*, 2013 ▸; Marinelli *et al.*, 2015 ▸).

An optics-based DXPG system, which is also called a split-and-delay optical (SDO) system, is highly useful for extending the delay range. A single XFEL pulse is divided into two pulses by a beam splitter. The split pulses propagate through two delay branches in the SDO system, and finally are recombined at a sample with a time delay. The delay time is determined by the path length difference between the delay branches, resulting in a jitter-free delay time control through precise adjustments of the path length difference. In the hard X-ray regime, several SDO layouts based on grazing-incidence multilayer mirrors (Roling *et al.*, 2014 ▸), diffraction gratings (David *et al.*, 2015 ▸) and perfect crystals (Roseker *et al.*, 2009 ▸, 2011 ▸; Stetsko *et al.*, 2013 ▸; Sakamoto *et al.*, 2017 ▸) have been proposed, and some systems have been tested at synchrotron and/or XFEL facilities. In particular, crystal-based SDO systems can easily generate a large delay time of more than 100 ps with a reasonably small apparatus, because of a large deflection angle (greater than several degrees) for Bragg diffractions. Furthermore, monochromatic XFEL pulses generated with the crystal-based SDO system are preferable for achieving high resolutions in typical X-ray diffraction experiments and for increasing the visibility of speckle patterns at a high scattering vector range in coherence-based experiments including XPCS. However, the crystal-based SDO systems that have been previously reported operate only at discrete photon energies, due to special optical configurations with high symmetry, such as 45° diffraction, 90° diffraction or multi-beam case diffraction. The development of an SDO system with wide and continuous photon energy ranges has been in high demand for broad applications such as resonance experiments of various elements.

We have developed a versatile SDO system operating in a wide and continuous photon energy range from 6.5 to 11.5 keV, and tested it at SPring-8 (Osaka *et al.*, 2016 ▸). In the test, a spectral division scheme with 10 µm-thick Si(220) crystals (Osaka *et al.*, 2013 ▸) was employed. Although the basic performance of this system was successfully confirmed, we found that a wavefront of the split beam reflected by the thin crystals was distorted, possibly because the lattice in the thin crystals were slightly bent due to the weakness of the structure against internal and external stresses. As a result, the focal profile in this thin-crystal branch was broadened. In this paper, we present the results of performance tests of the SDO system that uses a wavefront division scheme with an edge-polished thick crystal, conducted at an XFEL facility, SPring-8 Angstrom Compact free-electron LAser (SACLA; Ishikawa *et al.*, 2012 ▸). We characterized the beam properties of the SDO system, and investigated its capabilities for beam manipulation and diagnostics.

## 2. Conceptual design of the SDO system   

**Figure 1:**
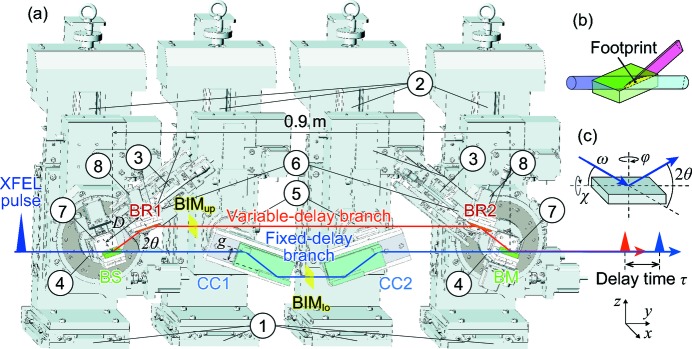
(*a*) Illustration of the mechanics of the SDO system. Crystals BS, BRs, BM and CCs represent the beam splitter, beam reflectors, beam merger and channel-cut crystals, respectively. Translation stages in the (1) *x*, (2) *z* and (3) *D* directions. Rotation stages around the (4) ω–2θ axis for BS/BM, (5) ω axis for CCs and (6) ω axis for BRs. Swivel stages around the χ axis for (7) BS/BM and (8) BRs. The range of *D* is set to 57–127 mm. Beam intensity monitors (BIM_up_ and BIM_lo_) are inserted into the two delay branches. Conceptual sketches of (*b*) wavefront division and (*c*) four general axes in X-ray diffraction.

Fig. 1(*a*) ▸ shows the schematics of the SDO system consisting of six separate Si(220) crystals in the vertical scattering geometry; a beam splitter (BS), two beam reflectors (BRs), a beam merger (BM) in the variable-delay (upper) branch, and a pair of speckle-free channel-cut crystals (CCs; Hirano *et al.*, 2016 ▸) in the fixed-delay (lower) branch. In the wavefront division scheme, an X-ray wavefront is laterally divided into reflected (upper) and unreflected (lower) beams at the edge of the BS, as illustrated in Fig. 1(*b*) ▸. The spectrum of each split beam can be independently selected, allowing for the production of double pulses with the same photon energies. Intensities of both branches could also be balanced because the split beams experience the same numbers of reflections with each other. Additionally, it is easy to control the intensity ratio by translating the edge positions of the BS and BM.

The mechanics of the SDO system [Fig. 1(*a*) ▸ and Table 1 ▸] followed those described by Osaka *et al.* (2016 ▸). For controlling the crystals in the upper branch, motorized precision stages for ω and χ axes [see Fig. 1(*c*) ▸] are utilized. A wide range of photon energies are covered continuously by changing the incident angle ω of the crystals. The photon energy range is 6.5–11.5 keV for the lower branch and above 3.2 keV for the upper branch.

The path length of the upper branch is controlled by translating the BRs along the beam axes with linear stages connected to the 2θ arms. The delay time τ is written as
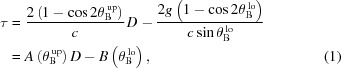
where *D* is the path length between adjacent BS/BM and BRs, 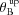 (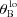) is the Bragg angle for the upper (lower) branch, *g* = 30 mm is the channel width of the CCs, and *c* is the speed of light. The coefficient *A* (*B*) is a function of the Bragg angle, *i.e.* the photon energy, for the upper (lower) branch. The translation range of the BRs was 57 < *D* < 127 mm. For example, the corresponding range of the delay time is −50 < τ < +47 ps at 10 keV (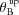 = 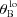 = 18.8°), while the maximum delay can be extended up to 220 ps at 6.5 keV (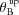 = 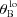 = 29.8°) in this system. The delay time is controlled with a resolution of <1 fs over the photon energy range of 6.5–11.5 keV.

## 3. Performance characterization   

We tested the SDO system at the hard X-ray beamline BL3 of SACLA (Tono *et al.*, 2013 ▸) to study the following targets: (i) Shot-to-shot non-invasive measurement of the pulse energies for both branches simultaneously. This capability is required for accurate analysis of experimental data to compensate possible fluctuation of XFEL pulse energies. (ii) Characterization of the focused beam profile of each branch. (iii) Spatial control of the relative beam positions on the focal plane. We note that precise angular tuning finer than 0.1 µrad is required for assuring an overlap between the focused beams in the present setup, as described in §3.2. (iv) Temporal control of the interval between two pulses, which is one of the most important targets of the SDO system, and can be characterized only with femtosecond XFEL pulses.

The experiment was performed with 10 keV monochromatic XFEL pulses through a Si(111) double-crystal monochromator (DCM) at a repetition rate of 30 Hz. The SDO system was installed ∼100 m downstream from the last undulator segment. The edge positions of the BS and BM were adjusted along the horizontal direction so as to transport the half portion of the incident beam profile into the upper branch. The exit beams from the SDO system were focused with a 1 µm focusing mirror system (Yumoto *et al.*, 2012 ▸) located 14 m downstream. Three inline diagnostic modules were implemented to measure the pulse energy of the incident beam (BIM0; Tono *et al.*, 2011 ▸) and those of the two delay branches (BIM_up_ and BIM_lo_) in the SDO system, as shown in Fig. 1(*a*) ▸. The modules BIM_up_ and BIM_lo_ consisted of a thin polyimide film scatter with a thickness of 50 µm, two photodiodes, and lead shields with ∼8 mm holes for XFEL transmission. They were also used as beam stoppers for a single branch operation mode by shifting their lateral positions. Pulse energies of the exit beams were measured with an optional PIN photodiode (PD_mirror_) placed in front of the focusing system. The delay time was measured with an X-ray streak camera (HAMAMATSU, C4575-03) placed 17 m downstream from the SDO system.

**Figure 2:**
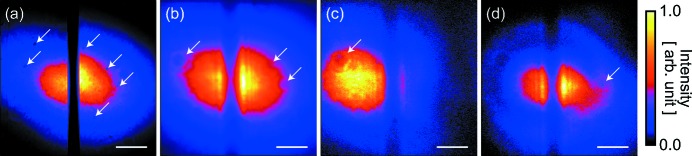
Averaged profiles of the exit beams measured with (*a*) BPM1 and (*b*) BPM2. The left (right) part of the profile in each panel shows the exit beam from the upper (lower) branch. Speckles as shown by the white arrows were caused by a Be window used for the upstream beamline optics and dust on the polyimide film of BIMs. (*c*, *d*) Typical single-shot beam profiles measured with BPM2. Differences between the profiles are due to the shot-to-shot profile and positional variations of the incident XFEL beams. The scale bars represent a length of 0.2 mm.

We coarsely adjusted the position and the angle of the upper beam by monitoring the beam positions at the detector planes of two beam profile monitors BPM1 (HAMAMATSU, ORCA flash with a 5.0× magnification lens system, 1.25 µm pixel^−1^) and BPM2 (4.0 µm pixel^−1^; Kameshima *et al.*, 2016 ▸), located 0.4 m and 16 m downstream of the SDO system, respectively. The incident angles ω of all crystals were precisely aligned to give the same photon energies for the upper and lower beams with an accuracy of <50 meV. Figs. 2(*a*) and 2(*b*) ▸ display averaged beam profiles measured by BPM1 and BPM2, respectively. We did not observe unwanted speckles from the crystals in the SDO system, whereas weak diffraction fringes from the edge of the BS and/or BM were obtained.

### 3.1. Shot-to-shot pulse energy diagnostics   

**Figure 3:**
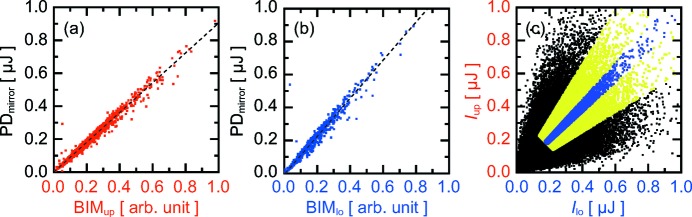
Shot-to-shot pulse energies of (*a*) upper and (*b*) lower branches for ∼1300 shots. The intensities measured with PD_mirror_ are plotted as a function of that with BIM_up_ and BIM_lo_ in (*a*) and (*b*), respectively. (*c*) Correlation between shot-to-shot pulse energies *I*_up_ and *I*_lo_ for 100000 shots. Yellow points (26% of the total events) satisfy the condition of the normalized difference |*R*_d_| = |2(*I*_up_ − *I*_lo_)/(*I*_up_ + *I*_lo_)| < 0.5 and *I*_up_ + *I*_lo_ > 0.35 µJ, while purple points (5.8% of the total events) show |*R*_d_| < 0.1 and *I*_up_ + *I*_lo_ > 0.35 µJ.

We investigated the feasibility of shot-to-shot non-invasive measurement of the pulse energies for the upper and lower branches simultaneously. For this purpose, we compared pulse energies measured with BIM_up_ (BIM_lo_) in the SDO system with those given by PD_mirror_ in the single branch operation mode, where the beam path of the lower (upper) branch was blocked. Excellent linearities were obtained for both cases, as plotted in Figs. 3(*a*) and 3(*b*) ▸. Errors from the fitted line were 6% in the relative standard deviation. The values measured with BIM_up_ (BIM_lo_) were converted to the pulse energies *I*_up_ (*I*_lo_) based on the fitting equation displayed as a dashed line in Fig. 3(*a*) ▸ (Fig. 3*b* ▸). Fig. 3(*c*) ▸ shows a correlation between *I*_up_ and *I*_lo_ for 100000 events. The ratio of pulse energies between the upper and lower branches was widely distributed shot-by-shot, whereas it was almost unity on average. The yellow points in Fig. 3(*c*) ▸ (26% of the total events) satisfy the condition of the normalized difference |*R*_d_| = |2(*I*_up_ − *I*_lo_)/(*I*_up_ + *I*_lo_)| < 0.5 and *I*_up_ + *I*_lo_ > 0.35 µJ, while the purple points (5.8% of the total events) show |*R*_d_| < 0.1 and *I*_up_ + *I*_lo_ > 0.35 µJ. The distribution originated from large shot-to-shot variations in profile and position of the incident Si(111) monochromatic beams as shown in Figs. 2(*c*) and 2(*d*) ▸. Note that the spectral fluctuations of the incident XFEL beam could not vary the ratio of the pulse energies, because both branches used the same photon energies in the wavefront division scheme.

The averaged pulse energies of the incidence beam and the sum of exit beams from both branches were 7.9 µJ and 0.38 µJ, respectively, indicating a throughput of 4.8%. Note that the throughput reflected not only an efficiency of the SDO system but also the transmissivities of the existing air pass of ∼2 m (∼30%) and the beamline components with additional polyimide windows on vacuum ducts (∼70%). Considering the total transmissivity of 20% measured with removing the SDO system, we evaluated the efficiency of the SDO system to be 24%, which was slightly smaller than the calculated value of 36%. The decrease in the measured efficiency may originate from slight angular errors on the optical elements and imperfection of the crystal lattice near the edges of the BS and BM.

### 3.2. Focusing characteristics   

**Figure 4:**
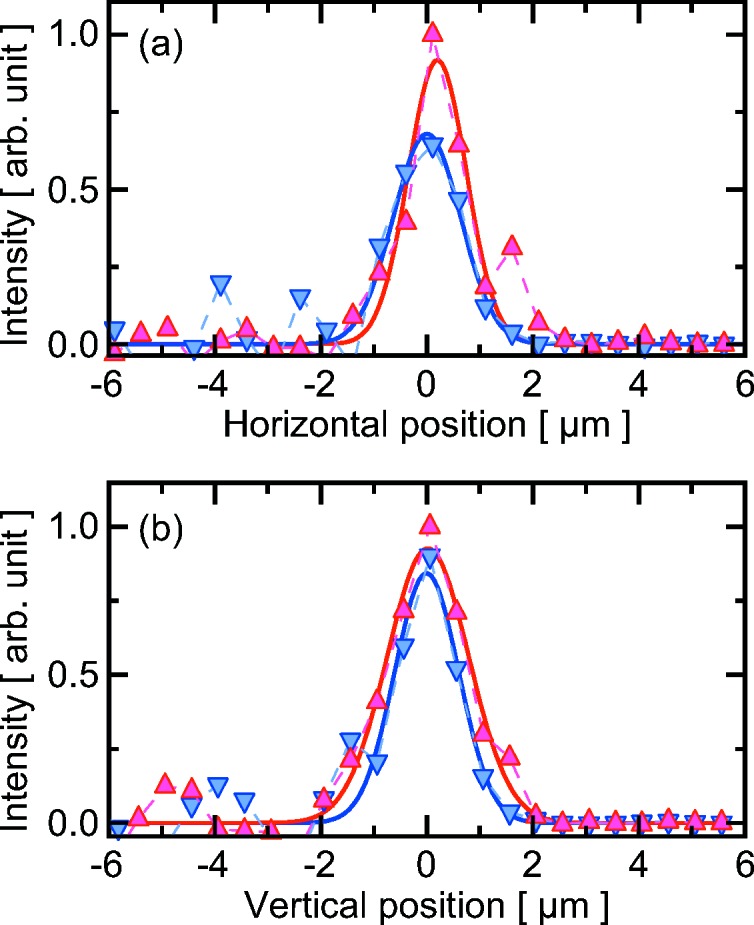
Averaged profiles of the focused beams in the (*a*) horizontal and (*b*) vertical directions. Red triangles and blue inverse triangles represent upper and lower branches, respectively. Each data point was measured using an average of ten shots. Solid curves indicate the fitted lines with the Gaussian functions.

An averaged focal profile of each branch was measured by the knife-edge scanning method, as shown in Fig. 4 ▸. The focal spot sizes of the upper and lower beams were 1.29 µm (H) × 1.81 µm (V) and 1.49 µm (H) × 1.42 µm (V) in full width at half-maximum, respectively, which almost agreed with those given in the ideal condition. This result showed that the wavefront distortion previously observed for the thin-crystal branch in the spectral division scheme was sufficiently reduced in the present case. Furthermore, Fig. 4 ▸ shows an excellent overlap of the two branches with an accuracy of <0.2 µm. This was accomplished by precise tuning of the angles of the crystals finer than 75 nrad and 0.23 µrad for the ω and χ axes, respectively, which was imposed by a typical source size of 60–80 µm and the distance between the source and the SDO system (Osaka *et al.*, 2016 ▸). Note that the focal positions and profiles vary shot-by-shot, which can be characterized by using a state-of-the-art high-resolution X-ray camera.

### 3.3. Delay time control   

**Figure 5:**
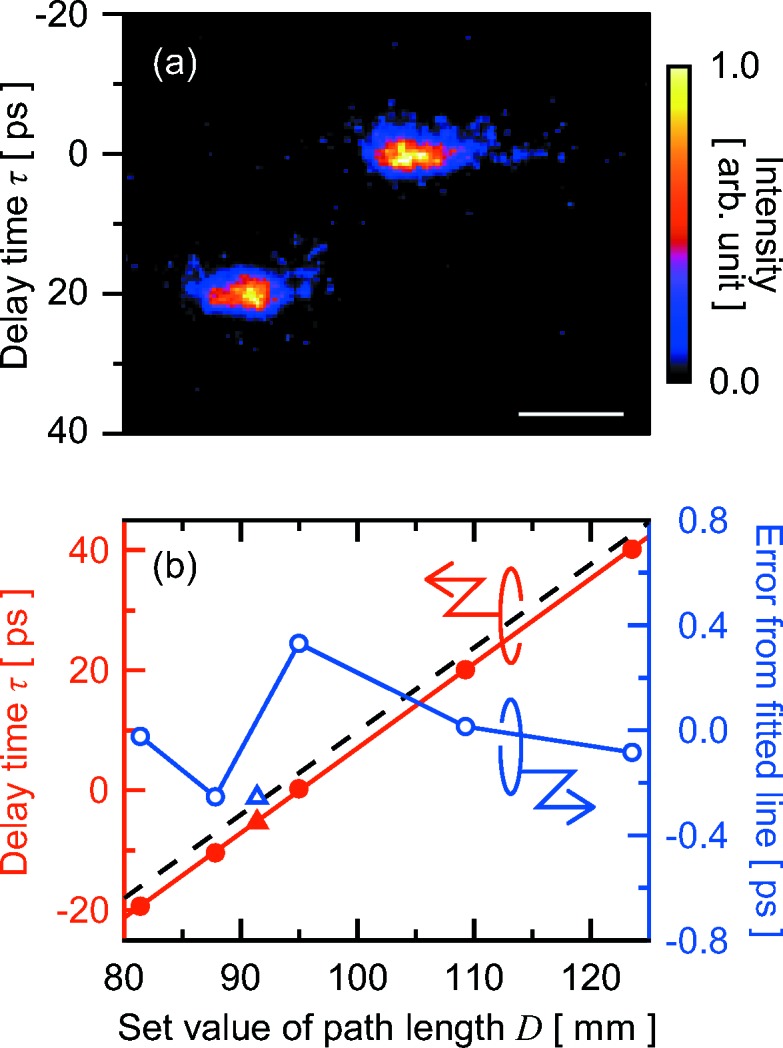
(*a*) Typical split-and-delayed pulses measured with the X-ray streak camera. The delay time between two pulses was set to be 20 ps. Profiles in the horizontal and vertical directions correspond to spatial (horizontal) and temporal information, respectively. The width of the temporal distribution of each pulse, ∼3 ps, was due to the effective resolution of the camera. The scale bar represents a length of 0.4 mm. (*b*) Delay time as a function of the setting path length between adjacent BS/BM and BRs. Averaged delay times at each set value of the path length are displayed as closed red circles. The solid red line represents the fitted line. Open blue circles indicate the errors from the fitted line. The dashed black line represents the calculated line in the ideal condition. Closed red and open blue triangles indicate averaged delay time and the error from the fitted line, respectively, measured after the delay scan plotted as circles.

We measured the delay time using the X-ray streak camera (Fig. 5*a* ▸). The temporal window, pixel resolution and effective time resolution under these experimental conditions were 200 ps, 0.47 ps and ∼3 ps, respectively. Averaged delay times between two pulses are plotted as a function of the setting path length *D* in Fig. 5(*b*) ▸. We obtained good linearity with a fitting error of ±0.5%. Errors from the fitted line were less than 0.4 ps over the time range of ∼60 ps. The slope values of the fitted and calculated lines based on equation (1) agreed with a small deviation of 1.0%. This error was explained by 0.07° angular deviations of both 2θ arms from the ideal positions. The offset of delay between measured and calculated values was ∼3 ps, which corresponds to the deviations in the setting path length *D* and in the channel width *g*.

## 4. Conclusion   

The performance tests of the SDO system with wavefront division scheme based on Si(220) diffraction of the perfect crystals, operating at a photon energy of 6.5–11.5 keV, were conducted with 10 keV XFELs at SACLA. We confirmed that shot-to-shot non-invasive pulse energy diagnostics for the two delay branches was feasible while the ratio of the pulse energies fluctuated strongly. We successfully obtained nearly ideal and identical focal beams with a spot size of ∼1.5 µm, with an excellent spatial superimposition in a sub-micrometer accuracy. The slope of the fitted line to the measured delay time as a function of the setting path length *D* was in good agreement with the calculated line.

We are planning to upgrade the SDO system with dedicated mechanics to enable more robust operation with reduction in the angular changes caused by the delay scan, while extending the range of the photon energy and the delay time. Also, the employment of an amplitude division scheme could generate double XFEL pulses with more identical properties without fluctuation of the ratio of the pulse energies, which requires ultrathin perfect Si(220) crystals with a thickness of 2 µm as BS and BM.

## Figures and Tables

**Table 1:** Stage resolutions in half-step feed mode

Number in Fig. 1(*a*) ▸	Crystal	Axis	Resolution
1	BS, BM, CCs	*x*	1.0 µm
2	BS, BM, CCs	*z*	1.0 µm
3	BRs	*D*	0.25 µm
4	BS, BM	ω	24.2 nrad
	BRs	2θ	34.9 µrad
5	CCs	ω	24.2 nrad
6	BRs	ω	0.70 µrad
7	BS, BM	χ	8.38 µrad†
8	BRs	χ	23.4 µrad

† The resolution in the χ axis of BS/BM was enhanced to 83.8 nrad by the operation in 1/200-step feed mode.
